# A qualitative study on tobacco use during the COVID-19 pandemic in Lebanon: Waterpipe and cigarette smokers’ views, risk perceptions, and behaviors

**DOI:** 10.18332/tpc/189770

**Published:** 2024-06-27

**Authors:** Rima Nakkash, Malak Tleis, Joanna Khalil, Maya Romani, Ramzi G. Salloum

**Affiliations:** 1 Global and Community Health Department, College of Public Health, George Mason University, Fairfax, Virginia, United States; 2 Department of Health Promotion and Community Health, Faculty of Health Sciences, American University of Beirut, Beirut, Lebanon; 3 Department of Family Medicine, Faculty of Medicine, American University of Beirut Medical Center, Beirut, Lebanon; 4 Department of Health Outcomes and Biomedical Informatics, College of Medicine, University of Florida, Gainesville, Florida, United States

**Keywords:** interviews, smoking behavior, pandemic, cigarettes, waterpipe

## Abstract

**INTRODUCTION:**

Since the beginning of the COVID-19 pandemic, a growing number of studies have documented more severe COVID-19 symptoms and worse outcomes among smokers compared to non-smokers. The aim of this research is to understand the views, risk perceptions, and behaviors of Lebanese adult smokers during the pandemic.

**METHODS:**

We conducted 18 qualitative online structured interviews with adults who smoke cigarettes and waterpipe tobacco residing in Lebanon from November 2020 through April 2021. Participants were recruited predominantly via paid social media ads. Interviews were audio-recorded using Zoom software then transcribed verbatim. Data were analyzed thematically.

**RESULTS:**

The findings showed three main themes: changes to smoking behaviors, concerns over the susceptibility and severity of COVID-19, and using coping methods to protect smokers from COVID-19. Although there was an increased risk perception of COVID-19 in relation to smoking, most participants reported an increase in smoking. Instead of being encouraged to quit, participants developed coping strategies against COVID-19 while smoking.

**CONCLUSIONS:**

Smoking behavior did not seem to decrease during the COVID-19 pandemic. To support cessation initiatives and raise awareness, effective health messaging aimed at smokers is pivotal. Smoking cessation programs need to be better equipped at supporting people who smoke in finding healthier coping mechanisms during a pandemic. Moreover, policies to regulate the propagation of misinformation are required to prevent the development of a false sense of safety and protection from COVID-19.

## INTRODUCTION

During the COVID-19 pandemic, several studies have examined the risk of smokers developing severe symptoms from COVID-19 and increased death rates compared to the general population ^1-4^. Quantitative and qualitative studies have evaluated the effect of the pandemic on smokers behaviors, perceptions, and attitudes toward smoking. In terms of smoking behavior, some countries reported an increase in smoking rates^5-7^, others a decrease^8-11^, some no change^12,13^, and others modifications in both directions within the same population^14-18^. Changes in smoking behavior during COVID-19 have been attributed to stress, susceptibility to infection, and increased time spent at home. For example, severely stressed smokers were more likely to increase or decrease their smoking regardless of their level of motivation to quit or perceived difficulty of quitting^15,19^.

While some studies reported a reduction in smoking as a result of mental distress caused by concerns of getting infected^19^, others reported increased smoking^7,20-22^. A qualitative study that conducted semi-structured individual interviews with 44 cigarette smokers and Electronic Nicotine Delivery Systems (ENDS) users in the United States found that increased tobacco use was predominant and driven mainly by individual-level factors (i.e. pandemic-related anxiety, boredom, and disruption of daily routines), while decreased use was common among social users who cited fewer interpersonal interactions (i.e. interactions or relationships with other people) and fear of sharing products^20^. A study in the UK found that smoking increased during lockdown as a result of more time spent at home, increased stress associated with confinement, curtailment of social routines, removal of barriers and distractions to smoking due to working from home, and feelings of boredom^22^. Boredom and increased time at home were also the reasons behind increased smoking among current smokers in a US study^21,22^. In Iran, a study investigating the perception of tobacco smoking during the pandemic reported that cigarette smokers were less likely to believe that their smoking behavior could spread the virus^23^. They also reported that waterpipe smokers believed that smoking has a protective effect against COVID-19 and leads to faster recovery in case of infection^23^.

Other studies found that motivation to quit was strongly associated with the perceived severity of COVID-19, concerns about developing severe illness^24,25^, the belief that smokers are at higher risk, stress, and support from close friends and family to quit^22,26^. To date, studies have reported that decreases in cigarette smoking were mainly observed among younger age groups^11,25,27^ and smokers with lower levels of nicotine dependence^9,10^. In Lebanon, an online survey noted a reduction in smoking after the lockdown among a non-representative sample of 948 young adults^28^.

Lebanon has suffered from weak enforcement of tobacco control policies for many years. The ratification of the World Health Organization’s Framework Convention on Tobacco Control by the Lebanese parliament in 2005 resulted in the issuance of Law 174 in 2011. This evidence-based tobacco control policy legislated a complete smoking ban in all closed public places, bans on advertising and marketing of tobacco products, and enforcement of larger textual and pictorial health warnings. Despite the presence of Law 174, weak compliance has meant that the prevalence of smoking remained largely unaffected^29^.

Although the impact of the COVID-19 pandemic on smoking behavior and patterns has been studied in various high-income countries, only one to date has been identified in a low-to-middle-income country; that country being Iran^23^, which has a different tobacco use profile and tobacco control policy framework than Lebanon. Assessing the impact of the COVID-19 pandemic in different contexts and cultures is an important step in further understanding smoking behavior. Consequently, the aim of this research is to understand the behaviors, risk perceptions, and views of adults who smoke with regard to tobacco use during the COVID-19 pandemic in Lebanon, a country with a high prevalence of tobacco use and a weak tobacco control policy framework. With this understanding, more evidence-based interventions can be developed and tailored to support adults who smoke in future pandemics.

## METHODS

### Study design and recruitment

The study, which is qualitative in nature, conducted in-depth interviews with adult smokers residing in Lebanon. The study utilized convenience sampling for recruitment of participants. Following the approval from the American University of Beirut’s Institutional Review Board, adults who smoke were recruited via paid social media ads published on X (formerly Twitter), Facebook, and Instagram over a three-month period. The ad included a survey link to a screener to confirm eligibility. The inclusion criteria included any participant able to complete the interview in English/Arabic; current cigarette and/or waterpipe smoker; and aged ≥18 years. Eligible participants were directed to the informed consent form where voluntary participation and confidentiality were emphasized and consent for interview recording was obtained. Those who consented were then able to complete a short background survey that included information on gender, age, education level, occupation, and current smoking behaviors. At the end of the survey, participants were asked for their contact information and to provide a convenient date and time for the interview. Follow-up was done via phone to confirm the interview date, and an email invitation was sent with the Zoom link. Although 39 individuals initially expressed interest in participating in the study, after follow-up, further eligibility criteria screening, and other external factors (e.g. no shows, participants changing their mind about participating), and upon reaching data saturation, the researchers finalized interviews with 18 participants (9 females and 9 males) (Table 1).

**Table 1:** Characteristics of participants, Lebanon, November 2020 – April 2021 (N=18)

*Characteristics*	*n*	*%*
**Gender**		
Female	9	50
Male	9	50
**Age** (years)		
18–27	10	55
28–37	5	28
38–47	3	17
**Education level**		
University graduate/Bachelor’s degree	11	61
Master’s degree/more	5	28
High school graduate	2	11
**Currently working**		
Yes	13	72
No	5	28
**Smoking status**		
Cigarettes only	10	55
Waterpipe only	5	28
Dual smokers	3	17

During the time between November 2020 and April 2021, eighteen interviews were conducted via Zoom video conferencing, each taking approximately 15–30 minutes. Interview questions involved open-ended questions which asked about the participant’s smoking history, followed by details on how their smoking behavior changed during and after the lockdown, what they thought about the risk of smokers of getting COVID-19 or presenting different symptoms than non-smokers, how the COVID-19 outbreak impacted their perception on the risks and/or benefits of smoking, as well as their previous and planned quit attempts. The interview guide is provided in the Supplementary file.

### Analysis

All 18 interviews were transcribed verbatim by a hired professional transcriber. Following transcription, thematic analysis was conducted by assigning codes and subcodes to the transcripts and then categorizing these codes/subcodes into themes. To ensure inter-rater reliability, one transcript underwent independent coding by three members of the research team. The three independent coding versions were compared, and differences discussed. This process helped validate the coding scheme and fine-tune the themes. Following the development, revision, and approval of the codebook by the research team, one researcher (MT) continued the coding of the remaining 17 transcripts.

Since data analysis was iterative (i.e. data was analyzed as interviews were being conducted), data collection stopped once saturation was achieved at 18 interviews.

## RESULTS

**Figure 1:**
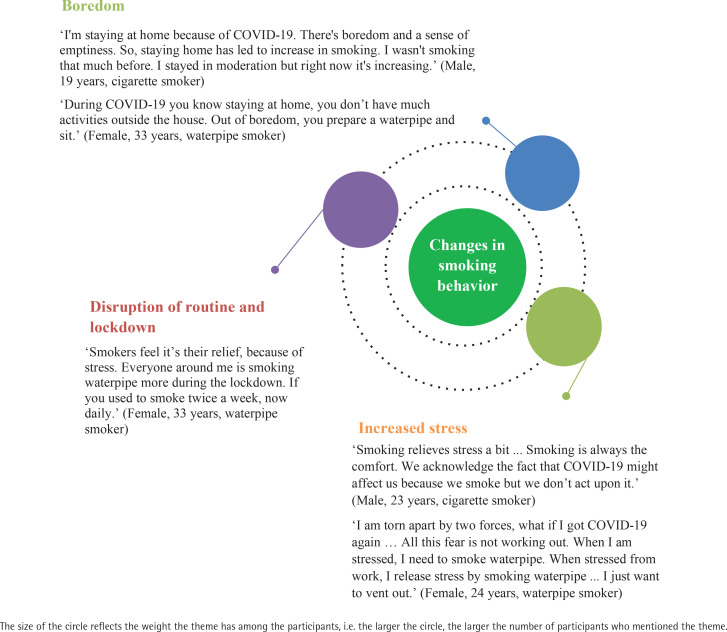
Quotes under the theme of ‘Changes in smoking behavior’

**Figure 2:**
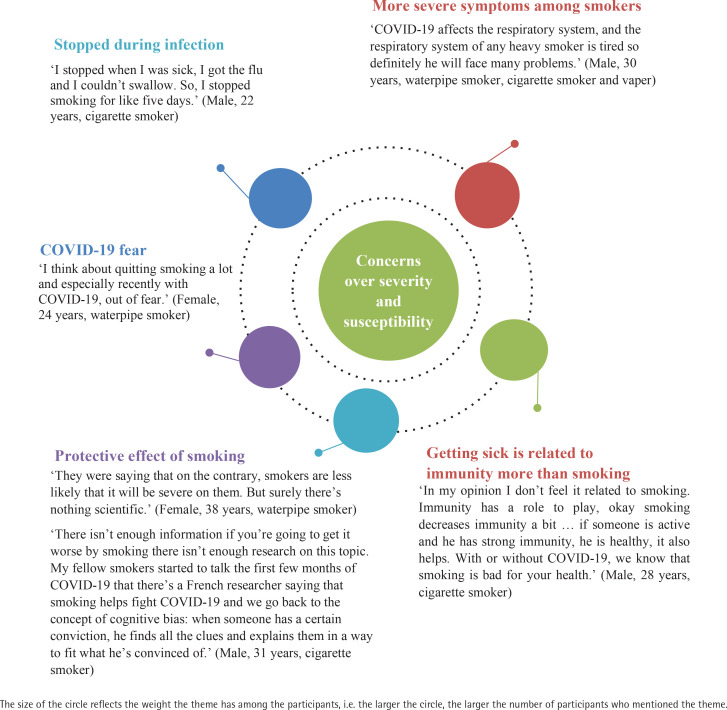
Quotes under the theme of ‘Concerns over severity and susceptibility’

**Figure 3:**
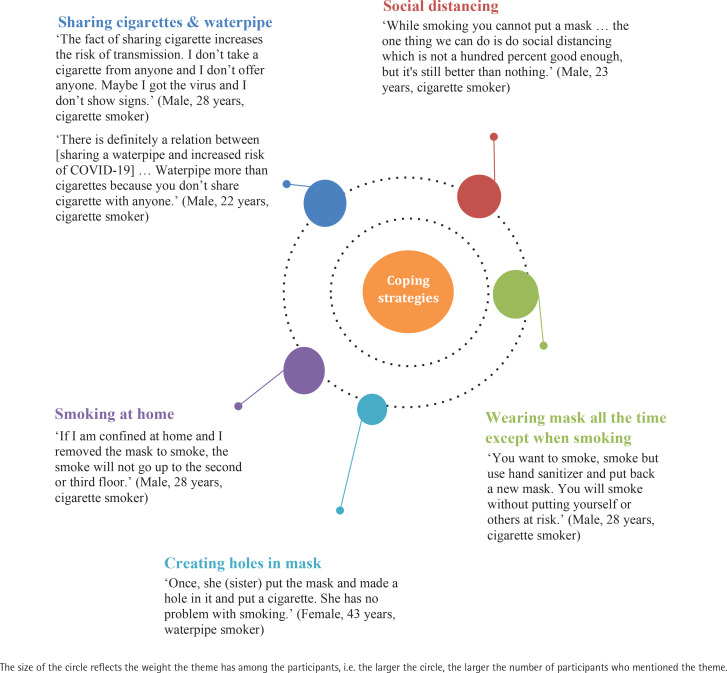
Quotes under the theme of ‘Coping strategies’

A total of 9 male and 9 females between aged 18–47 years participated in the study. More than half of the participants (61%) were currently enrolled in or had completed an undergraduate degree. In terms of smoking status, 55% of participants reported that they smoked exclusively cigarettes, 28% exclusively waterpipe, and 27% smoked both cigarettes and waterpipe. The main themes that emerged were related to changes in smoking behaviors and concerns over COVID-19, both in terms of susceptibility and severity of disease, and coping methods to protect smokers from COVID-19 while maintaining their smoking behavior at home, work, and in public places. These themes are presented below and illustrated with quotations (Figures 1–3).

### Reasons for changes in smoking behaviors during the COVID-19 pandemic

Ten out of 18 participants reported increased smoking due to boredom, ample free time, disruption of routine activities, stress, and lockdown. Many stated that if they had been at work or engaged in other activities, they would not have increased their smoking. This pattern was also reportedly observed amongst their friends and for the same reasons:

*‘Because of COVID, those of us who had work stayed at home … We want to pass the time with anything, so we pass the time with cigarettes and waterpipe unconsciously because we don’t have something else to do.’* (Male, 30 years, waterpipe smoker, cigarette smoker, and e-cigarette user)

The increase in smoking frequency and intensity was often notable:

*‘Consumption of cigarettes and waterpipe increased tremendously. Let’s say before COVID if I used to smoke one pack or one pack and a half, during COVID, I started smoking two packs, and I might smoke more, and there’s a possibility that at night … we put a waterpipe. So COVID increased tobacco consumption.’* (Male, 30 years, waterpipe smoker, cigarette smoker, and e-cigarette user)

Some smokers, however, reported that they smoked less due to imposed social distancing and lockdown measures. Those who were uncomfortable smoking around family members also decreased their smoking frequency or changed their smoking routine during lockdown. However, this decrease seemed temporary:

*‘… because of the quarantine with people who get bothered from smoking, yes, I decrease a bit and now came back like before.’* (Female, 24 years, cigarette smoker)

Masking primarily affected the frequency but not the intensity of their smoking:

*‘We’re wearing masks all the time; we definitely smoked less. In fact, imposing masks mainly affected our frequency of smoking. Actually, it didn’t make us decrease smoking because sooner or later we are catching up, but it affected the way we used to smoke’* (Male, 25 years, cigarette smoker)

### Concerns over severity and susceptibility to COVID-19

Responses varied among participants regarding the association between smoking and COVID-19. Some acknowledged an increased risk of infection or symptoms among smokers while others dismissed it. Nine out of the 18 participants did not believe there was an increased risk of COVID-19 and one-third were convinced that smoking had a protective effect against COVID-19:

*‘There’s a study that showed that nicotine would be sitting on the lungs where COVID-19 would sit, so when someone gets it, the virus doesn’t have a place to stick on the lungs. I read this news immediately on social media.’* (Male, 25 years, cigarette smoker) *‘If we want to talk in a non-scientific way, maybe smoking kills the virus, it is possible. I’m convinced that smoking kills humans, so it is possible that it kills the virus. From my humble knowledge, smoking makes like a layer inside the lungs that decreases the absorption of oxygen from the air; I think so, I’m not sure, but it might be the reason. COVID-19 enters and gets out of our body because it doesn’t stick to the lungs because it is already covered with smoking materials … Let’s say I have a 30–40% conviction that smokers are less likely to get COVID-19.’* (Male, 30 years, waterpipe smoker, cigarette smoker, and e-cigarette user)

Some based their opinions on experiences they witnessed of heavy and elderly smokers who got COVID-19 with mild symptoms. Seeing non-smokers and young people getting infected by COVID-19 also convinced them that smokers were not at higher risk than the rest of the population.

One prominent assumption was that the impact of COVID-19 varies depending on the individual’s immunity and not on smoking status. Another factor was the different strains of COVID-19, some with severe symptoms and others showing mild symptoms:

*‘Smoking decreases immunity, so the symptoms will increase and for the lungs too because if COVID-19 caused infection in the lungs, this is also a harder problem. So, if he smokes, he already has bad lungs, and it will get worse, and he might need oxygen.’* (Female, 22 years, cigarette smoker)

*‘What I’m convinced with is that COVID-19 virus has different strains. If you got the virus with severe symptoms, you would feel severe symptoms, whether I smoke or not, whether I have a health problem or not.’* (Male, 25 years, waterpipe smoker and cigarette smoker)

The majority of the participants did not modify their smoking patterns during the pandemic despite their friends and family discussing the increased risk of COVID-19 among smokers:

*‘We always used to discuss the risks of COVID with my friends and my parents. There is my sister, who doesn’t smoke waterpipe or cigarettes, she would tell me: “save your lungs, maybe you caught COVID so that you don’t get affected by the symptoms much”. Everyone says that cigarette and waterpipe smoking are very much related to more severe COVID symptoms.’* (Female, 33 years, waterpipe smoker)

Most of the participants affirmed that smokers are indeed at higher risk for severity of symptoms rather than at increased susceptibility to infection. The increased severity of infection was explained by the respiratory system being the common organ affected by both smoking and COVID-19:

*‘When I used to have the flu, I did not use to feel that tired. When I started smoking, I said: “no, this is too much”. Now I feel that smoking has a big effect on COVID symptoms. Smoking anyway causes a cough or mucus, so a smoker would anyway have mucus in his body, so when he gets COVID, it will all come out, and there is the problem … Smoking decreases immunity, so the symptoms will increase for the lungs too because if COVID causes infection in the lungs, this is also a harder problem. So if he smokes, he already has bad lungs, and it will get worse and he might need oxygen.’* (Female, 22 years, cigarette smoker)

Participants who noted the increased risk among smokers expressed fear of the virus. For others, the scare came from a close encounter with COVID-19; they knew smokers who passed away because of COVID-19 or they were in contact with COVID-19 positive cases. However, despite the expressed COVID-19 scare pronounced by smokers, this scare seemed to start with the onset of COVID-19 and then fade as participants gave up on certain precautions and reverted to their previous smoking behaviors:

*‘I almost reached a level of total despair from life, that’s it, ages are in God’s hands, we contract COVID-19, let it be! What can we do!’* (Male, 28 years, cigarette smoker)

To cope with the COVID-19 scare, half the participants discussed the importance of protective measures; however, those participants did not consider quitting smoking as a protective measure. The five participants who reported they had caught and recovered from COVID-19 noted having to stop smoking temporarily during peak symptoms.

Others mentioned that they decided to temporarily stop smoking while being sick with COVID-19 for a quicker recovery:

*‘… During that time (COVID-19 infection) … Two weeks I didn’t smoke any kind of tobacco. I couldn’t smoke waterpipe at first, and it is better for my health and to get rid of it fast enough. Yes, I felt it was not good for the throat, the heart, and the lungs. So, I decided to stop it during that period.’* (Female, 29 years, waterpipe smoker)

Four participants admitted that they stopped smoking not due to a personal decision (i.e. decisions or perceptions regarding COVID-19 and smoking which stem from factors that only impact the person themselves) or fear from COVID-19 complications, but because their bodies could not handle smoking while being sick:

Most of these participants returned to their smoking routine after recovery. Only one participant reported quitting smoking during COVID-19 and maintaining abstinence:

*‘When it happened, I couldn’t smoke, it is not that I was afraid of something, but my body couldn’t handle smoking. It couldn’t. It is always like this when I get sick.’* (Male, 23 years, cigarette smoker)

Only three participants reported that their families and peers perceived that there is an increased risk of COVID-19 on smokers, while six participants shared that their acquaintances were not concerned about COVID-19 at all. Furthermore, five participants seemed to avoid discussing the topic altogether with their family and friends:

*‘Honestly, I don’t think they are aware of [COVID-19]. The important thing for him is to go smoke waterpipe, to share the waterpipe or not, they don’t care.’* (Male, 22 years, cigarette smoker)

Participants did not seem to differentiate between cigarette and waterpipe smoking when it came to risks of contracting COVID-19 nor risk of worsened symptoms. The views regarding increased risk were mainly related to how heavy the smoking was, regardless of the means of smoking.

### Smokers’ coping strategies during the pandemic

Participants described several coping mechanisms to maintain smoking during COVID-19, which were specific to the location of smoking (home vs public place) or smoking mode (waterpipe vs cigarettes).

To avoid the risk of COVID-19 infection, many waterpipe smokers stopped sharing the same hose with others while smoking. They explained how the sharing of the waterpipe and the act of passing it from one person to another could transmit the virus:

*‘When I am visiting friends, I take my own waterpipe. I wasn’t even sharing with my sister. Each one has her own waterpipe. I don’t share waterpipe with anyone of my friends, not even my sister. I smoke my own waterpipe.’* (Female, 33 years, waterpipe smoker)

Also, sharing cigarettes and lighters was mentioned as sources of increased risk for COVID-19 transmission. With contaminated hands, sharing any product can pose a threat to smokers:

*‘One person in the office was COVID positive, and I was in contact with that person. How was I in contact? We share a lighter. Why? Why? So now I am no longer sharing lighters. But back then, I didn’t catch COVID, that person didn’t transmit COVID to me because he was asymptomatic. So, I think sharing cigarettes and lighters … sometimes I hold a cigarette in my hand and I pass it to someone … these behaviors pose extra risk honestly.’* (Female, 24 years, cigarette smoker)

Switching to smoking at home rather than in public places was the most mentioned coping measure adopted by cigarette and waterpipe smokers. Participants stated that they were able to compensate for what they missed during the day once they were home:

*‘I smoke more when I get home. I don’t smoke a lot [during the day], and then when I get home, I smoke to the level that I wanted to smoke when I was outside.*

*And I try not to go out and not go smoking as much as I used to go out and smoke. So yeah, that’s pretty much what I do.’* (Male, 30 years, cigarette smoker)

Participants mostly agreed that if an individual took all the necessary measures while away from home, smoking at home would not put them at risk of contracting COVID-19:

*‘When I come home at night and smoke with my husband, I feel safe. I put on the mask all the time at work, and till now, I didn’t contract COVID. It will not come from the waterpipe because I am not smoking outside, just at home and with my own cigarette. I am taking good precautions, and I am not interacting with anyone.’* (Female, 38 years, waterpipe smoker)

Along with the protective measures imposed by employers in the workplace, social distancing in smoking areas was mentioned as a main protective factor. Participants also mentioned taking shorter smoking breaks and smoking less during COVID-19 to decrease exposure to COVID-19:

*‘For example, I work in a mall. So, in the mall, there is a special place to smoke in, a smoking area. So, I try not to go up there as often as I used to before to smoke. I used to smoke at work every one hour. And now I’m trying to do it like every 2–2.5 hours to go and smoke.’* (Male, 30 years, cigarette smoker)

Furthermore, participants shared that they always wore their masks during working hours except during cigarette breaks. Those same participants recognized that removing the mask to smoke would cause significant increases in the risk of spreading or contracting COVID-19:

*‘If someone has COVID and he is smoking, he would spread the virus in the air, the droplets or anything … if someone has low immunity it will contract it. If there are 2 or 3 smoking together and one is infected, he will contaminate the others. The moment he removed the mask to smoke and vented out the smoke, the problem starts because if he is not smoking, he wouldn’t have removed the mask.’* (Male, 28 years, cigarette smoker)

Some smokers reported seeing other smokers who created a cigarette-sized opening in their masks so that they could smoke while still wearing their masks.

Participants were divided between those who initially preferred to smoke at home and others who mainly favored smoking in public places prior to the COVID-19 pandemic. Still, both groups agreed on the increased risks associated with smoking in public places. As a coping strategy, more than half of the participants affirmed that they stopped smoking in public places, specifically waterpipe smoking in cafés. Some participants were concerned about the carelessness of waterpipe smokers and how they overlooked protective measures. Both cigarette and waterpipe smokers expressed these apprehensions:

*‘That’s it, we are outdoors we are smoking, we removed our masks, and we come close to one another and we chat. But this is wrong! There is COVID-19 indoors but not outdoors?? Many people have this mentality, like yeah, it’s cool, lite up a cigarette and come sit next to me. No! I don’t want to sit next to you. It has been happening a lot among smokers in the last couple of months.’* (Male, 23 years, cigarette smoker)

## DISCUSSION

Disruptions to routine caused by the COVID-19 pandemic influenced smoking patterns, especially during the early lockdown stages or during infection with the virus^20^. Shifts in everyday activities and more time spent at home were the prevailing reasons behind increased smoking. Overall, participants continued to smoke despite their awareness of the health consequences of getting infected with COVID-19. To manage the situation, smokers adopted several coping strategies.

While participants reported increased smoking during lockdown, in contrast they reported reduced or complete cessation of smoking during infection or peak symptoms. This finding is echoed in a Serbian study which found that infected smokers were more capable of decreasing their tobacco use^30^. Furthermore, a few participants reduced their smoking due to imposed measures during lockdown, but the majority reverted to regular smoking behaviors and resumed their normal smoking frequency after the lockdown was lifted.

In most of the reviewed studies, COVID-19-related scares and health concerns were factors that led to reduced tobacco use. Increases in tobacco use were seen as a coping mechanism to deal with COVID-19 stress and other factors. In our study, stress contributed to a lack of will to reduce smoking, even when participants were aware of the health consequences of COVID-19. With the stress of confinement and fear of the pandemic, tobacco use became a coping strategy^31,32^.

While stress was the main reason for maintaining smoking during the pandemic, the prevailing reason for increased smoking was shifts in life routine^20-22^. The pandemic changed the structure of everyday life; increased time spent at home and feeling of boredom led to changes in smoking behavior, mainly increased tobacco use. These findings were also documented in similar studies ^20,22^.

When discussing the association between smoking and COVID-19 susceptibility, several participants conveyed that smoking had a protective effect against COVID-19. Albeit a surprising finding, it is concurrent with a similar study conducted in Iran where waterpipe smokers also mentioned the perceived protective effect of smoking against COVID-19^23^. These misconceptions may be linked to exposure to misleading information, mainly from social media, as reported by some participants. Nonetheless, there was a level of awareness that higher risk smokers might have been associated with severity of symptoms. Those who expressed COVID-19 concerns were more prone to it at the beginning of lockdown, but concerns faded away with time.

To avoid infection, smokers described several coping strategies. One of which was avoiding smoking in public – smoking at home rather than in public was also believed to be safe practice by participants in the Iranian study^23^. Waterpipe smoking is commonly done in social settings, this was minimized during lockdown as a precautionary measure. Sharing waterpipes, a common pre-pandemic practice in Lebanon to which there is strong evidence of it facilitating the transmission of infectious diseases^33^, was temporarily stopped during the pandemic. Some participants also mentioned not sharing cigarettes and lighters as a way to prevent contamination. Smokers tended to maintain social distancing in smoking areas at work, and others reported seeing people creating a hole in their masks to smoke.

Stress and boredom brought on by the pandemic and its subsequent impact on tobacco use among people who smoke calls for the need to allocate more resources to smoking cessation programs and tailored support to people who smoke during future pandemics of similar nature^5^. These programs could provide more support and information on adopting better-coping mechanisms in stress-inducing situations like the pandemic. Furthermore, the influence of social media in spreading misinformation on the associations between COVID-19 and smoking, has been an important finding in this study – illustrating the necessity of effective community awareness and media campaigns provided by credible sources (e.g. healthcare professionals/organizations) to dispel misinformation around COVID-19 and smoking. More anti-smoking campaigns highlighting the increased health risks that people who smoke face during a respiratory pandemic, coupled with evidence-based smoking cessation programs that are equipped at helping to manage stress and anxiety during a pandemic, could greatly reduce tobacco use among people who smoke ^5,30^.

### Limitations

Although the study adds important insights to the already available literature, some limitations are to be acknowledged. Conducting interviews online, while necessary during the pandemic, poses potential challenges in building rapport with participants. The ease with which rapport can be established through online interviews has been a point of contention in research, with literature showing conflicting findings^34^. In this study, with the unstable internet connection in Lebanon, some verbal or facial cues may have been missed thus impacting rapport with the participants. Furthermore, recruitment through social media could have potentially resulted in demographic selection bias, with the advert only reaching ‘a younger more internet-active population’^35^. And finally, as data collection started in November 2020, a time when Lebanon was no longer in lockdown for the pandemic, the study relied on participants’ memory, which might have resulted in participant recall bias.

Despite its limitations, the study has provided a better understanding of smoking behavior in the time of a pandemic, specifically in the context of Lebanon. Findings from this study should serve as a call to action for researchers to build and sustain much needed post-pandemic surveillance mechanisms that are able to document the changing patterns of tobacco use in low-resourced settings similar to that of Lebanon.

Moreover, with Lebanon’s weak tobacco control policies, this research, and others of its kind^36^, further articulate the need not only for more tobacco control policies but for a more stringent enforcement of these policies. With participants reporting that they reduced or stopped smoking during infection or peak symptoms has shown the impact of the pandemic, which should be utilized to strengthen cessation programs, as smokers seem to be more receptive to smoking cessation efforts during a pandemic^30,36^.

## CONCLUSIONS

Although the results reflected participants feeling scared and perceiving COVID-19 risk on the smoker, smoking behavior either remained unchanged or increased. Using tobacco to cope with stress took precedence over the fear of getting infected. Accurate information on the link between the severity of COVID-19 symptoms and smoking is needed. To mitigate the potential contributions of the pandemic, medical and public health workforces must implement multi-level, innovative strategies to support quitting and minimize harm among those who use tobacco products during this time and in the years ahead.

## Supplementary Material



## Data Availability

The data supporting this research are available from the authors on reasonable request.
