# Supplementary material for: A qualitative study on tobacco use during the COVID-19 pandemic in Lebanon: Waterpipe and cigarette smokers’ views, risk perceptions, and behaviors
Source: Tob Prev Cessat. 2024 Jun 27;10:10.18332/tpc/189770. doi: 10.18332/tpc/189770 (PMC11209738; doi:10.18332/tpc/189770)
Supplement: Supplementary file 1 [file TPC-10-27-s1.pdf]

Qualitative in-depth interview guiding questions

1. How has your cigarette use changed during COVID-19 outbreak? Has it increased? Decreased? Stayed the same? If it has increased or decreased, please tell us the reasons behind the change? What is influencing the change in your cigarette use?

2. How has your waterpipe use changed during COVID-19 outbreak? Has it increased? Decreased? Stayed the same? If it has increased or decreased, please tell us the reasons behind the change? What is influencing the change in your waterpipe use?

3. Do you use any other products?
   a. E-cigarette
   b. Cigar/cigarillo
   c. Chewing tobacco
   d. Midwakh
   e. Other:

4. How has your use of each of the other products changed? Has it increased? Decreased? Stayed the same? Please tell us the reasons behind the change in use in each product other than cigarettes or waterpipe?

5. What concerns or questions do you have about your use of cigarettes during the COVID-19 outbreak?

6. What concerns or questions do you have about your use of waterpipe during the COVID-19 outbreak?

Institutional Review Board
American University of Beirut
02 SEP 2020
APPROVED

7. What concerns or questions do you have about your use of other tobacco products during the COVID-19 outbreak? (Choose just one product – e-cigarettes)

8. How has the COVID-19 outbreak changed your perception about the risks or benefits of using cigarettes? Probes: Do you think smoking cigarettes is more dangerous to your health during COVID- 19 – Why or Why not?

9. How has the COVID-19 outbreak changed your perception about the risks or benefits of using waterpipe? Probes: Do you think smoking waterpipe is more dangerous to your health during COVID- 19 – Why or Why not?

© 2024 Nakkash R. et al.

Institutional Review Board
American University of Beirut
0 2 SEP 2020
APPROVED
